# Sleep quality and COVID-19 outcomes: the evidence-based lessons in the framework of predictive, preventive and personalised (3P) medicine

**DOI:** 10.1007/s13167-021-00245-2

**Published:** 2021-06-08

**Authors:** Kneginja Richter, Stefanie Kellner, Thomas Hillemacher, Olga Golubnitschaja

**Affiliations:** 1 Outpatient Clinic for Sleep Disorders, University Clinic for Psychiatry and Psychotherapy, Paracelsus Medical University Nuremberg, 90419 Nuremberg, Germany; 2 Faculty for Social Work, Technical University of Applied Sciences Nuremberg Georg Simon Ohm, 90489 Nuremberg, Germany; 3 Faculty for Medical Sciences, University Goce Delcev Stip, 2000 Stip, North Macedonia; 4 Predictive, Preventive and Personalised (3P) Medicine, Department of Radiation Oncology, University Hospital Bonn, Rheinische Friedrich-Wilhelms-Universität Bonn, 53127 Bonn, Germany

**Keywords:** Predictive preventive and personalised medicine (PPPM/3PM), COVID-19, SARS-CoV-2, ICU, Sleep quality, Sleep duration, Sleep disturbance and deprivation, Sleep–wake rhythm, Insomnia, Depression, Anxiety, Gender, Pneumonia, Disease progression, Patient stratification, Individual outcomes, Complications, Modifiable risk factors, Risk assessment, Shift workers, Healthcare givers, Melatonin, Treatment, Drug, Comorbidities, Immune response, Anti-inflammation, Education, Health policy

## Abstract

Sleep quality and duration play a pivotal role in maintaining physical and mental health. In turn, sleep shortage, deprivation and disorders are per evidence the risk factors and facilitators of a broad spectrum of disorders, amongst others including depression, stroke, chronic inflammation, cancers, immune defence insufficiency and individual predisposition to infection diseases with poor outcomes, for example, related to the COVID-19 pandemic. Keeping in mind that COVID-19-related global infection distribution is neither the first nor the last pandemic severely affecting societies around the globe to the costs of human lives accompanied with enormous economic burden, lessons by predictive, preventive and personalised (3P) medical approach are essential to learn and to follow being better prepared to defend against global pandemics. To this end, under extreme conditions such as the current COVID-19 pandemic, the reciprocal interrelationship between the sleep quality and individual outcomes becomes evident, namely, at the levels of disease predisposition, severe versus mild disease progression, development of disease complications, poor outcomes and related mortality for both - population and healthcare givers. The latter is the prominent example clearly demonstrating the causality of severe outcomes, when the long-lasting work overload and shift work rhythm evidently lead to the sleep shortage and/or deprivation that in turn causes immune response insufficiency and strong predisposition to the acute infection with complications. This article highlights and provides an in-depth analysis of the concerted risk factors related to the sleep disturbances under the COVID-19 pandemic followed by the evidence-based recommendations in the framework of predictive, preventive and personalised medical approach.

## Preamble

Keeping in mind that COVID-19-related global infection distribution is neither the first nor the last pandemic severely affecting societies around the globe to the costs of human lives accompanied with enormous economic burden, lessons by predictive, preventive and personalised medical (PPPM/3PM) approach are essential to learn and to follow being better prepared to defend against global pandemics. To this end, 3PM knowledge towards the COVID-19 pandemic is regularly updated in the literature [1, 2]. Unique research data towards individual outcomes in COVID-19-affected patient cohorts accompanied by potent predictive and preventive approaches have been presented and acknowledged as a great contribution of 3P medicine to the global challenge by COVID-19 [3–5].

This article focused on the comprehensive sleep quality impacts, follows the series of publications presenting valuable 3PM lessons towards the COVID-19 relevant knowledge. Sleep quality plays a pivotal role in maintaining physical and mental health. In turn, sleep shortage, deprivation as well as sleep disorders are per evidence the risk factors and facilitators of a broad spectrum of disorders, among others including depression, stroke, chronic inflammation, cancer, immune defence insufficiency and individual predisposition to infection diseases with poor outcomes. Therefore, sleep deficits are highly relevant for the COVID-19 distribution and outcomes.

## Up-to-date facts and working hypotheses

### Sleep quality and immune defence

Sleep quality and duration have been shown to play a pivotal role in immune health. Accumulated research data clearly indicate that the immune defence is supported by sleep, whereas sleep deprivation, e.g. caused by insomnia and circadian disruption, can severely impair the immune system functionality serving therefore, as a reliable predictor [6, 7]. During the sleep, there is an increased activity of two major subtypes of lymphocytes, which are important for the adaptive immune response and general disease prevention, namely, CD4 + “helper” T cells and cytotoxic CD8 + “killer” T cells. Further, a decreased activity of natural killer cells and increased production of pro-inflammatory cytokines are important for the host defence against pathogens including viral infections [7–13]. During night-time sleep, pro-inflammatory hormones and cytokines are synchronised to facilitate the onset of an adaptive immune response. In contrast, during the daytime activity, anti-inflammatory signals, hormones and cytokines are supportive for immediate reactions towards biological and other environmental challenges [14]. Additionally, the cytokines TNF-α and IL-12 as well as antigen-presenting dendritic cells (DCs) show their highest activity during the night in those adhering to a normal sleep–wake routine, but also in those kept awake for 24 h, and without change of the period (24 h) of these rhythms. The peak time of the circadian rhythms of cortisol, epinephrine, norepinephrine and the cytokine IL-10 occurs early in the morning, around the time of awakening from night-time sleep according to the individual sleep–wake rhythm. In situations of continuous wakefulness, epinephrine displays an undefined rhythm. The peak time of the rhythm in IL-10 shifts to the night-time. Cortisol and norepinephrine show peak value rhythms similar to sleep values, which Nadir values, for both, are higher under conditions of constant wakefulness [15]. Also reduced activities of natural killer cell activity and T cell cytokine production in sleep-deprived subjects compared to subjects with a full night of sleep have been demonstrated [16, 17].

In this context, the relationship between sleep quality and proper immune defence functionality becomes particularly relevant for the COVID-19 pandemic outcomes. Indeed, tiredness occurs as one of the common symptoms in the early stages of COVID-19 infection, and as the diseases progresses, insomnia may emerge as an additional problem, with disturbed sleep persisting beyond the acute stage of the disease. In terms of possible long-term effects of COVID-19 that exceed the typical recovery period, bundled under the term long COVID-19 or post-acute sequelae of SARS-CoV-2 infection, effects on sleep and fatigue are apparent, with sleep problems and fatigue becoming typical symptoms of long COVID-19 6 months after infection. In the context of long COVID-19, Huang et al. reported in a sample of N = 1655 that 437 individuals (26%) showed sleep problems and fatigue or muscle weakness for 1038 individuals (63%) [17].

Among its multi-faceted functions, sleep has been shown to regulate glucose metabolism and weight gain, both of which are acknowledged risk factors of diabetes, obesity and sleep apnoea, which in turn have been associated with a higher predisposition to disease on viral infections [18] and to severe COVID-19 disease progression [19, 20].

### Sleep quality and vaccination efficacy

Considering the importance of vaccination as the primary strategy to combat viral pandemics, research data provide clear evidence towards an important role of the sleep quality in the level of vaccination efficacy [21]. It has been reported that a sufficient amount of deep and slow-wave sleep could be essential for vaccine effectiveness, as the common flu vaccination seems to be more effective in people who have slept well in the days preceding the vaccination [22]. Another study conducted by Lange et al. examined the role of sleep and wakefulness after receiving hepatitis A vaccination on immune responses. Results have shown that after sleep compared to wakefulness, the relative portion of the virus-specific Th cells were doubled, and the portion of Th1 cytokine-producing cells increased being important for an adaptive immune response [23]. Contextually, research data on the relationship between the sleep quality and the vaccination efficacy as well as the relevance of the impaired sleep are of great importance.

### Sleep quality-improving medication and COVID-19 outcomes

While there are no specific antiviral therapeutic agents available for the COVID-19 yet [24, 25], sleep-related treatment options improving individual outcomes are available. To this end, Zhou et al. demonstrated that coronavirus infection can lead to insomnia resulting in a disturbed production of melatonin with adverse effects on the immune defence and long-term pathologic alterations in the nervous systems [26]. Based on the collected statistics, this research group hypothesised that the COVID-19 replication could be inhibited by the intake of melatonin [26].

A retrospective analysis performed by Columbia University indicated improved survival rates for the intubated ICU patients, if they were treated with melatonin [27]. To this end, Cheng et al. suggest the melatonin function in the sleep quality. In addition to its key role in regulating circadian rhythms, melatonin also modulates the immune system by inhibiting inflammation and cytokine storm as one of the most dangerous components of the COVID-19 disease [28]. Indeed, melatonin acts as anti-inflammatory and immune-modulating agent effectively boosting human immune system [22]. The review of Mohamed et al. summarises evidence on the utility of melatonin as a potential antioxidant adjuvant in COVID-19-infected individuals with diabetes and obesity, suggesting melatonin as a potent treatment option to improve COVID-19 outcomes by supporting immune health [29]. To date, there is no direct experimental evidence on the virucidal effect of melatonin; however, research data demonstrate indirect activities of melatonin as a potent antiviral helper, due to its complex anti-inflammatory, antioxidant and immunomodulatory properties [30, 31].

### Poor sleep quality predicts a susceptibility to infections in healthcare gives

Healthcare givers, among the shift workers in general, are particularly susceptible to impaired sleep, due to rotating working hours and night work. Shift work, especially night work, induces a disruption of the circadian rhythm compromising the neuroimmune-endocrine homeostasis including a reduction in natural killer cells activity that has been well documented for nurses [32–37]. Sleep rhythm disturbance characteristic for the shift workers is clearly associated with a higher vulnerability to infectious diseases such as a flu [38–40], whereas reduced sleep (< 5 h) and poor sleep quality and/or excessive sleep (> 9 h) are linked with a higher risk for pneumonia [41, 42]. Further, sleep-deprived shift workers are at higher risk of respiratory infections and their severity grad [43, 44] that is highly relevant for the susceptibility to the COVID-19 infection and related complications. The higher risk and severity of respiratory and other infections may thus not only be associated with sleep disorders, but also to a reduced amount of slow-wave NREM sleep, which is responsible for the secretion of the cytokine IL-12 and DCs, the main precursors of Antigen-presenting cells [45, 46].

An epidemiological survey conducted in December 2019 in Wuhan revealed the highest prevalence of the poor sleep quality in 18.2% of the sample (N = 7236) specifically for healthcare givers demonstrating compared to all other professional occupations [47]. In particular, healthcare givers working on the frontline with the acutely COVID-19 diseased individuals are directly exposed to the pathogen accompanied with the sleep imbalance and circadian disruption that makes them to a special group of risk which well-designed predictive and preventive medical approach should be applied for to protect against the health damage linked to their professional duties [48].

### Working hypothesis in the context of PPPM

Based on the above presented facts, a reciprocal interrelationship has been hypothesised between the sleep quality and individual outcomes for both COVID-19 diseased and medical staff involved in the care. If being valid, this concept opens great prospects for implementing risk assessment, patient stratification, individualised prediction and targeted prevention to persons at high risk among professional and in the population linked to the well-justified educational measures.

### Literature search methodology

Systematic literature search has been conducted utilising electronic data available in PubMed and Google Scholar.

Literature search investigated effects of COVID-19 on a sleep quality in hospital populations, separately considering both — the medical staff and affected patient cohorts.

Studies dedicated to the sleep quality of healthcare givers were identified by following combinations of the keywords, “COVID-19”, “sleep quality”, “medical staff”, “sleep disturbances” and “insomnia”. The review was limited to published research articles written in English. Only original research articles about or literature reviews of the effects of sleep on medical staff with reported sleep assessments as outcome variables were included.

Studies dedicated to the sleep quality of the affected patients were identified by following combination of the keywords, “COVID-19”, “patients”, “sleep”, “sleep quality” and “ICU”. The review was limited to published research articles written in English. Only original research articles or literature reviews on the effects of COVID-19 on patients’ sleep were included. Furthermore, only studies measuring sleep status were included.

**Fig. 1:**
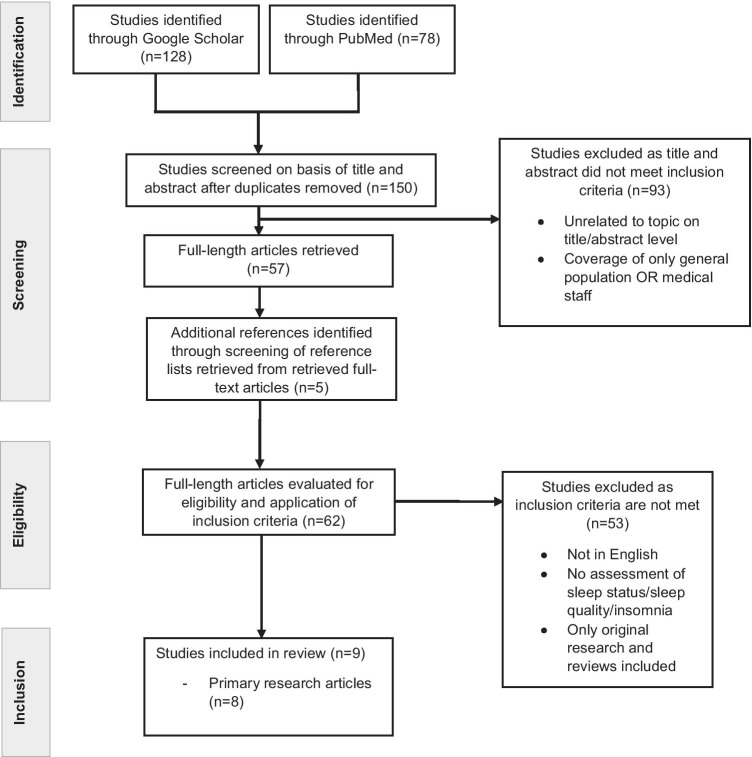
Flowchart according to the PRISMA criteria on sleep disorders in COVID-19 patients

**Fig. 2:**
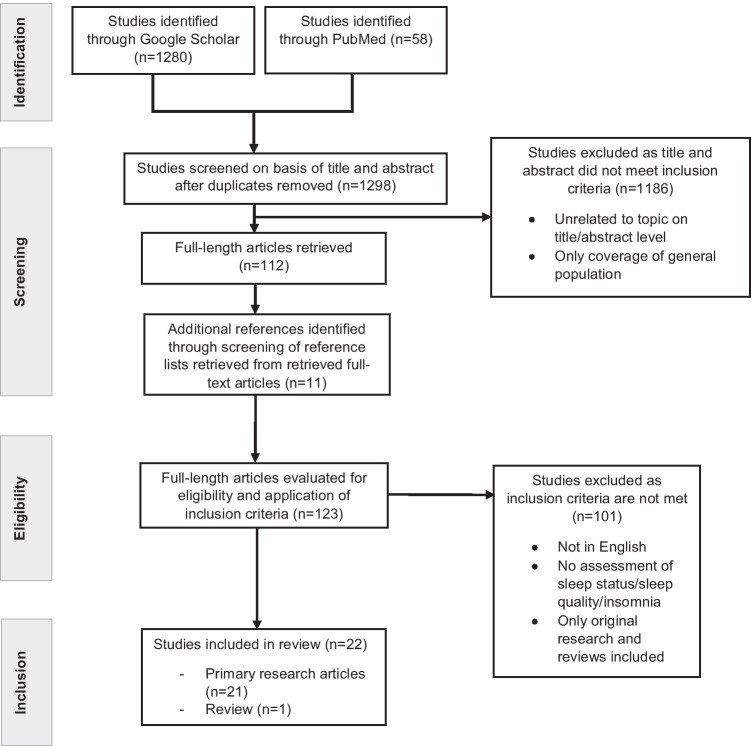
Flowchart according to the PRISMA criteria on sleep disorders in healthcare providers under the COVID-19 pandemic condition

Both search approaches are summarised in Figs. 1 and 2, respectively.

## Data interpretation

### Sleep quality in the COVID-19-affected patients

Table 1 presents the collected data with corresponding interpretation towards analysed sleep quality and related aspects in the COVID-19-affected patients. Analysed studies clearly demonstrate that insomnia symptoms persist in 36–88% of all included COVID-19 patients — the level which is significantly higher compared to the prevalence of insomnia in the general population estimated as being 10 to 40% [49, 50]. The underlying causes of the poor sleep parameters are considered as being:

1. Potentially related to early awakening by medical staff and disrupted sleep
2. Due to sedative administration
3. Due to mechanical ventilation of affected COVID-19 patients [51].

**Table 1:** Sleep disorders in the COVID-19 patient cohorts

Author, year	Title	Sample size (N)	Sample (date)	Country	Study design	Data interpretation
Dai, Wang et al., 2020 [52]	Anxiety and depressive symptoms among COVID-19 patients in Jianghan Fangcang Shelter Hospital in Wuhan, China	307	Hospitalised COVID-19 patients (February 2020)	China	Cross-sectional study	Prevalence of anxiety, depressive symptoms and poor sleep quality: 18.6%, 13.4% and 84.7%, respectively, poor sleep quality and having $\ge$ two physical symptoms as risk factors for anxiety symptoms (p $<$ 0.05), female sex, cases of COVID-19 in family and $\ge$ two physical symptoms as risk factors for depressive symptoms (p $<$ 0.05)
Jiang, Zhu et al., 2020 [53]	Psychological distress and sleep quality of COVID-19 patients in Wuhan, a lockdown city as the epicenter of COVID-19	202	Hospitalised COVID-19 patients (February to March 2020)	China	Cross-sectional study	Gender as an independent predictor for anxiety (p $<$ 0.05) and depression status (p $<$ 0.05), association of lower education levels and little subjective knowledge of COVID-19 with higher depression scores (p $<$ 0.05), association of frequency of contacting with family with lower depression scores (p $<$ 0.05), association of subjective evaluation of disease symptoms and evaluation of medical staff’s attitude with mental distress assessments (p $<$ 0.05), association of age and evaluation of disease symptoms with sleep quality scores (p $<$ 0.05)
Li, Zheng et al., 2020 [54]	Rehabilitation needs of the first cohort of post-acute COVID-19 patients in Hubei, China	280	Hospitalised COVID-19 patients (February to March 2020)	China	Cross-sectional study	Main physical dysfunctions displayed by patients: sleep disorders (63.6%), decreased activity endurance (61.4%) and respiratory dysfunction (57.9%), main psychological anxiety (62.1%), fear (50.0%), apathy (41.8%) and depression (40.7%), main rehabilitation demands of patients: exercise guidance (45.0%), dietary instruction (40.4%) and traditional Chinese medicine therapy (39.6%)
Liu, Baumeister et al., 2020 [55]	Risk factors associated with mental illness in hospital discharged patients infected with COVID-19 in Wuhan, China	675	Discharged COVID-19 patients (April 2020)	China	Cross-sectional study	Main adverse mental health issue after hospitalisation, sleep disorders; further mental health problems, anxiety (10.4%) and depression (19%); central predictor of mental illness, perceived discrimination associated with the social stigma of COVID-19
Liu, Chen et al., 2020 [56]	Effects of progressive muscle relaxation on anxiety and sleep quality in patients with COVID-19	51	Hospitalised COVID-19 patients (January to February 2020)	China	Randomised controlled clinical trial	Progressive muscle relaxation as an effective auxiliary method to reduce anxiety and improve sleep quality in patients with COVID-19
Nalleballe, Reddy Onteddu et al., 2020 [57]	Spectrum of neuropsychiatric manifestations in COVID-19	40,469	COVID-19 patients (January to June 2020)	USA	Cross-sectional study	22.5% patients had neuropsychiatric manifestations. Most common neurologic manifestations, headache (3.7%) and sleep disorders (3.4%), encephalopathy (2.3%), stroke and transient ischemic attack (TIA) (1.0%); most common psychiatric manifestations, anxiety and other related disorders (4.6%) and mood disorders (3.8%)
Vitale, Perazzo et al., 2020 [51]	Is disruption of sleep quality a consequence of severe Covid-19 infection? A case-series examination	4	Hospitalised COVID-19 patients in ICU (April to May 2020)	Italy	Case study	Subjective sleep complaints among COVID-19 ICU patients (mean PSQI score of 6.0 ± 1.22) and insufficient duration of sleep (potentially attributed to forced early awakening by medical staff); attenuated sleep quality and disrupted sleep habits might be due to administration of sedative medications needed during mechanical ventilation
Zhang, Xu et al., 2020 [58]	Poor-sleep is associated with slow recovery from lymphopenia and an increased need for ICU care in hospitalized patients with COVID-19: A retrospective cohort study	135	Hospitalised COVID-19 patients (January to March 2020)	China	Retrospective, single-centre cohort study	Association between poor sleep quality during hospitalisation in COVID-19 patients with lymphopenia with a slow recovery from lymphopenia (p $<$ 0.05), an increased need for ICU care (p $<$ 0.05) and increased duration of hospital stay (p $<$ 0.05)

Poor sleep quality was consequently associated with the lower levels of lymphocytes in the blood of COVID-19 patients [58]. To this end, deep sleep modulates the T-Help cell response that plays a key role in the immune response towards viral infections suggesting that a focus on improving sleep quality in COVID-19 patients may be decisive for the recovery.

Further, an evident gender difference has been observed: female patients suffered more frequently from poorer psychiatric outcomes, such as depression and anxiety, compared to the male patients [52, 53].

To improve sleep quality in COVID-19 patients, Liu et al. suggest implementing progressive muscle relaxation, for which anxiety-relieving effects were also reported [54].

### Sleep quality in the healthcare givers under the COVID-19 pandemic condition

Table 2 presents the collected data with corresponding interpretation towards analysed sleep quality in healthcare givers. The analysed data indicate that working with COVID-19-affected patients is associated not only with a poorer sleep quality and insomnia symptoms, but also with mental health risks such as increased stress and burnout as well as a strong predisposition to mental disorders such as depression and anxiety [56, 57, 59–77].

**Table 2:** Sleep disorders in healthcare givers treating COVID-19-affected patients

Author, year	Title	Sample Size	Sample (date)	Country	Study design	Data interpretation
Alshekaili, Hassan et al., 2020 [59]	Factors associated with mental health outcomes across healthcare settings in Oman during COVID-19: frontline versus non-frontline healthcare workers	1139	Healthcare workers (HCW) (April 2020)	Oman	Cross-sectional study	During the pandemic period in April, 368 (32.3%), 388 (34.1%), 271 (23.8%) and 211 (18.5%) respondents were reported to have depression, anxiety, stress and insomnia, respectively; frontline HCWs were 1.5 times more likely to report insomnia (OR = 1.586, p = 0.013), anxiety (OR = 1.557, p = 0.004) and stress (OR = 1.506, p = 0.016)
Araç, Dönmezdil 2020 [60]	Investigation of mental health among hospital workers in the COVID-19 pandemic: a cross-sectional study	198	Healthcare workers treating COVID-19 patients	Turkey	Cross-sectional study	PSQI subscores and perceived stress levels were significantly higher among the volunteers working in the emergency department than among those in other departments ( p < 0.01); risk of development of anxiety among women was 16.6 times higher than among men
Ballesio, Lombardo et al., 2020 [61]	Caring for the carers: Advice for dealing with sleep problems of hospital staff during the COVID-19 outbreak	-	-	-	Review	Central health issues for frontline workers, sleep problems; association of sleep deprivation, long shifts and insomnia in hospital staff with increased risk of mental and somatic disorders, altered immune responses, medical errors, misunderstandings, drowsy driving and burnout; practical advice on sleep problems for hospital staff is to be provided
Barua, Zaman et al., 2020 [62]	Psychological burden of the COVID-19 pandemic and its associated factors among frontline doctors of Bangladesh: a cross-sectional study	370	Frontline doctors treating COVID-19 patients (April to May 2020)	Bangladesh	Cross-sectional study	Among the doctors, 36.5% were reported to have anxiety, 38.4% had depression, 18.6% displayed insomnia symptoms, and 31.9% had fear of COVID-19; inadequate resources in the workplace as the single most significant predictor for all psychological outcomes, anxiety and/or depression (severe, OR 3.0, p = 0.01; moderate, OR 5.3, p = 0.000; mild, OR 2.3, p = 0.003), sleep disturbance (moderate, OR 1.9, p = 0.02) and fear of COVID-19 (severe, OR 1.9, p = 0.03; moderate, OR 1.8, p = 0.03)
Herrero, Parra-Serrano et al., 2020 [63]	Sleep characteristics in health workers exposed to the COVID-19 pandemic	170	Frontline HCWs and non-HCWs (March to April 2020)	Spain	Cross-sectional study	Self-reported insomnia, nightmares, sleepwalking, sleep terrors and lower subjective sleep quality were more frequent in the healthcare group (all p < 0.05)
Jahrami, BaHammam et al., 2020 [64]	The examination of sleep quality for frontline healthcare workers during the outbreak of COVID-19	257	Frontline HCWs and Non-HCWs (April 2020)	Bahrain	Cross-sectional study	Poor subjective sleep and moderate-high levels of stress were reported by both groups (60%); female sex and professional background were the predictors of poor sleep quality and stress
Kang, Ma et al., 2020 [65]	Impact on mental health and perceptions of psychological care among medical and nursing staff in Wuhan during the 2019 novel coronavirus disease outbreak: A cross-sectional study	994	HCWs (January to February 2020)	China	Cross-sectional study	Among the medical and nursing staff during the immediate epidemic, 36.9% had subthreshold mental health disturbances, 34.4% had mild disturbances, 22.4% had moderate disturbances, and 6.2% had severe disturbance (mean PHQ-9, 15.1); young women were reported to be at higher risk; 36.3% had accessed psychological materials (such as books on mental health), 50.4% had accessed psychological resources available through media (such as online push messages on mental health self-help coping methods), and 17.5% received counselling or psychotherapy
Liang, Wu et al., 2020 [66]	Mental Health in Frontline Medical Workers during the 2019 Novel Coronavirus Disease Epidemic in China: A Comparison with the General Population	2003	HCWs and non-HCWs (February to March 2020)	China	Cross-sectional study	Of the frontline medical workers, 30.43%, 20.29% and 14.49% of frontline HCWs in Hubei Province and 23.13%, 13.14% and 10.64% of frontline HCWs in other regions reported symptoms of depression, anxiety and insomnia, respectively. Of the general population, 23.33%, 16.67% and 6.67% of the general population in Hubei Province and 18.25%, 9.22% and 7.17% of the general population in other regions reported symptoms of depression, anxiety and insomnia, respectively
Liu, Jiang et al., 2020 [67]	The effectiveness of diaphragmatic breathing relaxation training for improving sleep quality among nursing staff during the COVID-19 outbreak: a before and after study	140	HCWs (February to March 2020)	China	Quasi-experimental intervention study	By practising diaphragmatic breathing relaxation: significant reductions in global sleep quality (p < 0.01), subjective sleep quality (p < 0.001), sleep latency (p < 0.01), sleep duration (p < 0.001), sleep disturbances (p < 0.001), habitual sleep efficiency (p < 0.015), daytime dysfunction (p < 0.001), and anxiety (p < 0.001) in frontline HCWs
Qi, Xu et al., 2020 [68]	The evaluation of sleep disturbances for Chinese frontline medical workers under the outbreak of COVID-19	1306	HCWs (February 2020)	China	Cross-sectional study	Among frontline HCWs: higher prevalence of sleep disturbances (p < 0.001) and worse sleep quality (p < 0.001), as well as reported anxiety (p < 0.001) and depression (p < 0.001) compared to non-frontline HCWs
Şahin, Aker et al., 2020 [69]	Prevalence of Depression, Anxiety, Distress and Insomnia and Related Factors in Healthcare Workers During COVID-19 Pandemic in Turkey	939	HCWs (April to May 2020)	Turkey	Cross-sectional study	Overall: 77.6% participants exhibited depression, 60.2% anxiety, 50.4% insomnia and 76.4% distress symptoms; females, frontline work, being tested positive for COVID-19, individuals with a history of psychiatric illness and individuals in psychiatric treatment were at greater risk for depression, anxiety, insomnia and distress symptoms
Stojanov, Malobabic et al., 2020 [70]	Quality of sleep and health-related quality of life among health care professionals treating patients with coronavirus disease-19	83	HCWs	Serbia	Cross-sectional study	Low quality of sleep and poor health-related quality of life were correlated with high health anxiety and severe depressive symptoms; higher scores of anxiety (p < 0.01) and lower scores on mental health (p < 0.01) as independent predictors for low subjective quality of sleep and self-rated depression;
Tu, He et al., 2020 [71]	Sleep quality and mood symptoms in conscripted frontline nurse in Wuhan, China during COVID-19 outbreak: A cross-sectional study	100	Frontline HCWs (February 2020)	China	Cross-sectional study	A total of 76%, 81%, 45% and 19% reported difficulty initiating sleep, difficulty maintaining sleep or early morning awakening, nightmares and using hypnotics, respectively. Among the HCWs, 60% reported poor sleep quality, 46% suffered depression symptoms, and 40% exhibited anxiety symptoms. Sleep quality (OR = 3.16, 95% CI, 1.17–8.52) and anxiety symptoms (OR = 8.07, 95% CI, 2.92–22.33) were significantly associated with depression symptoms. Depression symptoms (OR = 3.24, 95% CI, 1.19–8.79) were associated with poor sleep quality
Wang, Zhang et al., 2020 [72]	Psychological impact of coronavirus disease (2019) (COVID-19) epidemic on medical staff in different posts in China: A multicenter study	274	HCWs (February to March 2020)	China	Multicentre cross-sectional study	Total scores of anxiety, depression, sleep quality and stress were statistically different among HCWs in Hubei and outside Hubei; increased risk of exposure to COVID-19 was linked to an increased tendency of anxiety, depression and lower subjective sleep quality (p < 0.05); HCWs in Hubei had the highest prevalence of anxiety (20%), depression (22%) and insomnia (26%); non-frontline HCWs outside Hubei had the lowest prevalence of anxiety (7.4%), depression (4.4%) and insomnia (10.3%) (adjusted p < 0.05); the combined prevalence of anxiety, depression and insomnia of staff in Hubei was estimated to be 38%; among the participants, 69.4% may need psychological support
Wang, Song et al., 2020 [73]	Sleep Disturbance and Psychological Profiles of Medical Staff and Non-Medical Staff During the Early Outbreak of COVID-19 in Hubei Province, China	2001	HCWs (March 2020)	China	Cross-sectional study	Among the HCWs, 61.6%, 35% and 22.6% reported sleep problems, depressive symptoms and anxiety, respectively; higher prevalence of sleep disorders for frontline HCWs compared to non-frontline and non-medical staff; significant predictors for poor sleep quality, medical occupation, family burden, bereavement, anxiety and depression
Wu, Wei et al., 2020 [74]	Analysis of Psychological and Sleep Status and Exercise Rehabilitation of Front-Line Clinical Staff in the Fight Against COVID-19 in China	120	HCWs	China	Cross-sectional study	Higher scores of somatisation, depression, anxiety and terror in frontline HCWs ( p < 0.05); frontline HCWs reported lower subjective sleep quality ( p < 0.05); medical staff who exercised according to the exercise recommendations reported better psychological stress and higher sleep quality
Xiao, Zhang et al., 2020 [75]	The Effects of Social Support on Sleep Quality of Medical Staff Treating Patients with Coronavirus Disease 2019 (COVID-19) in January and February 2020 in China	180	Frontline HCWs (January to February 2020)	China	Cross-sectional study	Significant positive association between levels of social support of HCWs and self-efficacy and sleep-quality ( all, p < 0.01); social support was negatively correlated with anxiety and stress (all, p < 0.01); anxiety, stress and self-efficacy as mediating variables associated with social support and sleep quality
Zhan, Liu et al., 2020 [76]	Factors associated with insomnia among Chinese front-line nurses fighting against COVID-19 in Wuhan: A cross-sectional survey	1794	Frontline HCWs (March 2020)	China	Cross-sectional study	Prevalence of insomnia among participants, 52.8%; predictors for insomnia, gender, working experience, chronic diseases, midday nap duration, exposure to COVID-19 patients, frequency of night shifts, psychological support, negative personal experiences due to COVID-19, degree of fear of COVID-19, fatigue and perceived stress
Zhang, Yang et al., 2020 [77]	Survey of Insomnia and Related Social Psychological Factors Among Medical Staff Involved in the 2019 Novel Coronavirus Disease Outbreak	1563	HCWs (January to February 2020)	China	Cross-sectional study	Insomnia symptoms were reported by 36.1%; associated with insomnia symptoms, (lower) education level (p < 0.05), occupation as a doctor (p < 0.01), exposure to COVID-19 (p < 0.05), fear of COVID-19 infection (p < 0.001), perceived lack of psychological support (p = 0.001), uncertainty regarding effective disease control (p < 0.05)
Zhang, Shi et al., 2020 [78]	Posttraumatic stress disorder symptoms in healthcare workers after the peak of the COVID-19 outbreak: A survey of a large tertiary care hospital in Wuhan	642	HCWs (June 2020)	China	Cross-sectional study	Prevalence of probable PTSD symptoms, 20.87%; among HCWs with PTSD, varying degrees of anxiety, depression, somatic symptoms and insomnia were reported by 88.88%, 82.09%, 100% and 95.52%, respectively; HCWs with probable PTSD had higher scores on depression scale (HADS), patient health questionnaire (PHQ-15) and insomnia (ISI) than non-PTSD HCWS; protective factors against probable PTSD, testing negative for COVID-19 (p < 0.01), perceived sufficient social support ( p < 0.01), family members tested negative for COVID-19 ( p < 0.05)
Zhou, Wang et al., 2020 [79]	The prevalence and risk factors of psychological disturbances of frontline medical staff in china under the COVID-19 epidemic: Workload should be concerned	1705	Frontline HCWs and non-HCWs (February to March 2020)	China	Cross-sectional study	The prevalence of depression, anxiety, somatisation symptoms, insomnia and suicide risk in frontline HCWs were 57.6%, 45.4%, 12.0%, 32.0% and 13.0%, respectively; among frontline HCWs, daily working hours were associated with all psychological disturbance (all, p < 0.01) and female sex was associated with anxiety (p < 0.05), body mass index with anxiety and insomnia (both, p < 0.05); age was negatively associated with depression, anxiety and insomnia (all, p < 0.01)
Zhuo, Gao et al., 2020 [80]	Stress and sleep: a survey based on wearable sleep trackers among medical and nursing staff in Wuhan during the COVID-19 pandemic	26	HCWs with insomnia symptoms (March 2020)	China	Cross-sectional study	Among participants, 38.5% demonstrated moderate to severe sleep apnoea–hypopnea syndrome (SAHS); comorbid moderate to severe SAHS was linked to higher scores on insomnia and worse mental health status (both, p < 0.05); insomnia symptoms were negatively correlated with deep sleep (p < 0.05); male sex was identified as a potential risk factor for insomnia with comorbid SAHS (OR = 11.56)

In agreement with earlier demonstrated sex differences in the sleep quality [81, 82], female sex is associated with poorer sleep outcomes. The collected data indicate that female healthcare givers working in shifts during the pandemic are at significantly higher risk for insomnia and other mental health disturbances. In consensus with previous studies dedicated to the sleep quality for the shift workers, female nurses are at increased risk to develop sleep disorders and to a cancer predisposition. In summary, working in shifts with the COVID-19-affected patients significantly increases overall risk of adverse effects to nurses’ health.

## Conclusions and expert recommendations in the framework of 3P medicine

### Being frontline healthcare givers under COVID-19 pandemics

Data collected clearly demonstrate that particularly frontline healthcare givers reported worse sleep outcome variables, such as insomnia symptoms or significantly decreased sleep quality accompanied by increased levels of stress and burnout as well as increased appearance of psychiatric symptoms such as depression and anxiety. Indeed, under the pandemic condition, the frontline healthcare givers are facing sudden outbreak, which given the severity of the pandemic leads to the work overloads and extended working time frame with insufficient time to recover and consequent chronic exposure to stress, psychological distress [47] and increased vulnerability to the stress-related disorders including viral infections.

Sleep deprivation in shift workers is associated with a higher incidence and severity of respiratory infections compared to non-shift workers [43, 44], suggesting that shift workers are more susceptible to COVID-19 infection and, consequently, to severe disease outcomes. In consensus, being dependent on the individual circadian rhythms, the adaptive and innate immune systems are disrupted by working in shifts. This in turn increases susceptibility of the affected individuals to infections [10, 38–40, 83]. To this end, the analysed molecular mechanisms argue in favour of a severe impairment of the immune memory in shift workers.

However, stress response and stress-related predisposition to pathologies have been demonstrated as being highly individual. Contextually, for working in shifts, an application of predictive diagnostic and patient stratification approaches followed by the targeted preventive measures is strongly recommended utilising corresponding tools and recommendations developed for a population screening and identification of individuals in sub-optimal health conditions [84, 85] and insomnia [86].

Contextually, perceived social and psychological support is considered as the preventive strategy to mild poor sleep quality and psychiatric symptoms in healthcare givers treating the COVID-19-affected patients [74, 75, 77, 78]. Recent studies on insomnia and sleep disorders related to working in shifts further indicate good effects for Internet-based interventions and conclude that hospital authorities are well advised to offer this new approach to their staff as part of workplace health promotion [87].

### Being COVID-19-affected

Analysed data clearly demonstrate that insomnia symptoms persist in big portion of the COVID-19 patient cohort with the monitored prevalence which significantly exceeds the level of insomnia in the general population. Several underlying causes have been proposed. Poor sleep quality was further associated with the decreased immune system functionality in the COVID-19 patients [58], suggesting that a focus on improving sleep quality may be decisive for the recovery and significantly better individual outcomes of the disease. Further, an evident gender difference indicates that particularly for the female patients, the focus on psychiatric aspects such as frequent depression and anxiety is strongly recommended as an essential part of individualised anti-COVID-19 treatment algorithms.

### Generalised anti-COVID-19 prevention

As the generalised anti-COVID-19 prevention, the evidence-based periodontal healthcare is strongly recommended [5].

To this end, periodontopathic microflora is implicated in systemic inflammation and pneumonia development, in severe cases leading to sepsis and death. Diagnosed periodontitis is associated with high risk of admission to intensive care units and increased COVID-19-related death [88]. To this end, educational measures play a pivotal role, conducting principles of participatory medicine as an essential element of 3PM and promoting an active participation of patients in the treatment procedure and taking great responsibility for their health status [5].

Taking into account multi-faceted anti-inflammatory and anti-mitochondriopathic effects of melatonin, melatonin treatment is strongly recommended as an essential pillar of the anti-COVID-19 protection for the shift workers, COVID-19-affected individuals and patients suffering from chronic non-communicable disorders under the viral pandemic conditions [89–91].

## Data Availability

Not applicable.
